# Fermented Soy Products: Beneficial Potential in Neurodegenerative Diseases

**DOI:** 10.3390/foods10030636

**Published:** 2021-03-18

**Authors:** Chan Ho Jang, Jisun Oh, Ji Sun Lim, Hyo Jung Kim, Jong-Sang Kim

**Affiliations:** 1 School of Food Science and Biotechnology, Kyungpook National University, Daegu 41566, Korea; cksghwkd7@gmail.com; 2 Institute of Agricultural Science and Technology, Kyungpook National University, Daegu 41566, Korea; j.oh@knu.ac.kr (J.O.); lzsunny@daum.net (J.S.L.); 3 Department of Korean Medicine Development, National Institute for Korean Medicine Development, Gyeongsan 38540, Korea; indersee31@nikom.or.kr; 4 Department of Integrative Biotechnology, Kyungpook National University, Daegu 41566, Korea

**Keywords:** fermented soybean products, Parkinson’s disease, Alzheimer’s disease, isoflavones, gut microbiota

## Abstract

Fermented soybean products, such as *cheonggukjang* (Japanese *natto*), *doenjang* (soy paste), *ganjang* (soy sauce), and *douchi*, are widely consumed in East Asian countries and are major sources of bioactive compounds. The fermentation of cooked soybean with bacteria (*Bacillus* spp.) and fungi (*Aspergillus* spp. and *Rhizopus* spp.) produces a variety of novel compounds, most of which possess health benefits. This review is focused on the preventive and ameliorative potential of fermented soy foods and their components to manage neurodegenerative diseases, including Alzheimer’s and Parkinson’s diseases.

## 1. Introduction

Population aging is a global demographic trend. According to the “2019 Revision of World Population Prospects” [1,2], the proportion of people aged 65 years or over worldwide is projected to reach nearly 16% by 2050, and 23% by 2100. However, this extended life expectancy is closely associated with a vulnerability to age-related disorders, such as neurodegenerative diseases, including Alzheimer’s disease (AD) and Parkinson’s disease (PD) [3]. Unfortunately, there is currently no effective treatment for these devastating diseases. Recently, an increasing number of studies have focused on the development of dietary measures as well as preventive regimens for these conditions [4,5]. For instance, the root of *Angelica gigas* and *Platycodon grandiflorus*, as well as *Lactobacillus helveticus* and phosphatidylserine, have been approved regarding the claim of cognition improvement by the Korean government, which allowed them to be processed and marketed as health functional foods.

The nutritional values and medicinal effects of soybean or its constituents are well documented [6,7,8,9,10,11]. In several Asian countries, including China, Indonesia, Japan, and Korea, fermented soybean products, such as *doenjang* (soybean paste and Japanese *miso*), *ganjang* (soy sauce), *natto*, and *tempeh*, have been extensively consumed since ancient times. Numerous studies published in the past decades have revealed that fermented soy products have multifarious health benefits, such as serum cholesterol-lowering, anti-diabetic, anti-hypertensive, anti-cardiovascular, and anti-neuroinflammatory effects [10,12,13,14]. Recently, soybean and its fermented products have received much attention regarding their effects on the gut microbiota, which are linked to the pathogenesis of various neurological disorders, including depression, anxiety, autism, AD, and PD [15,16,17].

This review article discusses the protective effects of popular fermented soy foods and their components in the context of neurodegenerative diseases, with a focus on AD and PD, and describes the possible mechanisms underlying the beneficial effects of these foods.

## 2. Types of Fermented Soy Products

**Figure 1:**
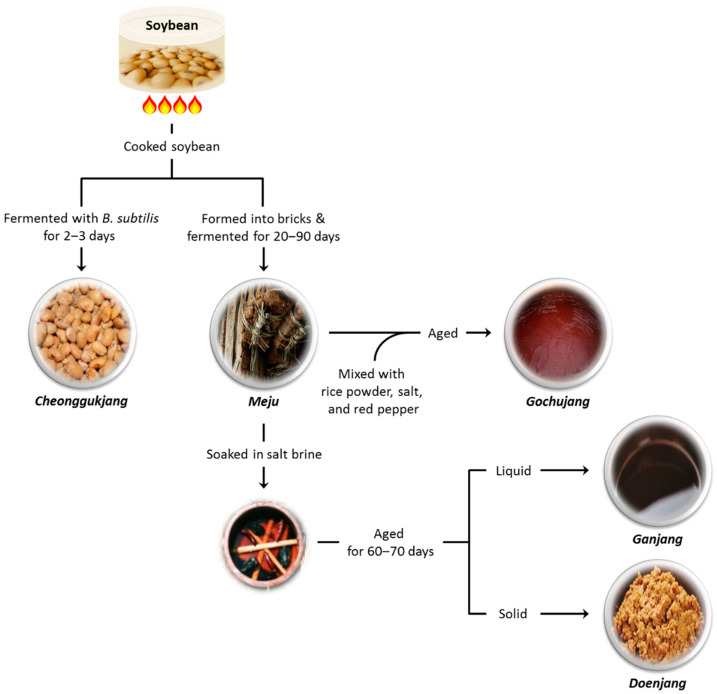
Examples of Korean fermented soy products.

Soybean has been processed into numerous types of products, such as soymilk, tofu, sprouts, and fermented products (Figure 1). The fermented products of soybean include *doenjang* (soybean paste), *ganjang* (soy sauce), Korean *cheonggukjang*, Japanese *natto*, Korean *gochujang*, Indonesian *tempeh*, *sieng* (Cambodia, Laos), *pepok* (Myanmar), *thua nao* (Thailand), and *knema* (India, Nepal, and Bhutan) [18].

Korean *cheonggukjang* and Japanese *natto* are both produced via two main steps, i.e., cooking and fermentation. In the first step, soybean is soaked in water at room temperature for 18 h, followed by steaming at 121 °C for 30 min. The second step consists in the fermentation of cooked soybean with airborne *Bacillus* species, including *Bacillus subtilis*, originating from the ambient environment or from inoculation for 48 h (Figure 1) [19].

While most of these products, including *cheonggukjang* and *natto*, are fermented with *Bacillus* spp., some products, such as *doenjang*, soy sauce, and *tempeh*, are manufactured by fermenting cooked soybean with fungi, such as *Aspergillus* and *Rhizopus*, resulting in the extensive breakdown of soy components and the production of novel bioactive compounds [18].

*Douchi*, which is a traditional Chinese food that is prepared using fermented and salted black soybeans, has been a popular seasoning in foods and a folk medicine in China for centuries [20]. *Sufu* or *furu* is one of the fermented soybean products, which has been consumed as a side dish in China over the centuries [21,22]. *Sufu* is a cheese-like product that is made by *Aspergillus oryzae* in solid-state fermentation of salted and ripened tofu through activities of hydrolytic enzymes, such as protease, α-amylase, β-amylase, and lipase [21,22].

## 3. Bioactive Components of Fermented Soy Products

Soybean contains a variety of biologically functional components that can be grouped into isoflavones, soyasaponins, lignans, cinnamic acid derivatives, terpenes, and sterols. The fermentation process results in the chemical modification and reduction of soy components. Although soybean is known to contain anti-nutritional factors, such as phytates, trypsin inhibitors and lectins [23], most fermented soy products have been analyzed to contain very small amounts of these factors, when compared with raw soybean [24]. In particular, lactic acid bacteria (LAB)-mediated fermentation can reduce phytates and trypsin inhibitors [25], and hydrolyze tannic acid via their tannase activities [26,27]. Almost all lectins in soybeans are destroyed during fermentation processes over 72 h [28]. Moreover, various novel compounds are generated during the fermentation process not originally present in raw soybean (Table 1).

**Figure 2:**
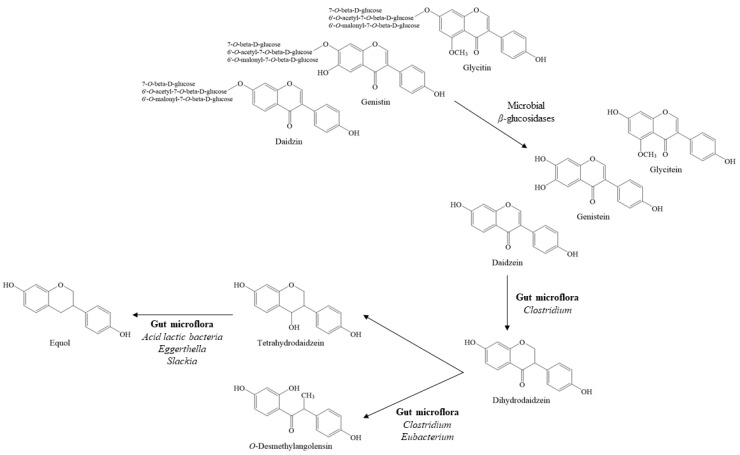
The biotransformation of soy isoflavones by microorganisms during food fermentation or in the gut.

The fermentation of cooked soybean with *Aspergillus* and other microorganisms, as performed during the manufacture of *meju* (a brick of dried fermented soybeans), generates novel compounds as well as extensively converts isoflavone glycosides into aglycones (Figure 2). For instance, free isoflavones account for 2.67% of the total isoflavones of soy flour, whereas aglycones represent more than 75% of the total isoflavones present in the product after fermentation for 48 h with *Aspergillus oryzae* [32]. In addition, extended fermentation was reported to reduce the amount of both aglycones and glycosides, although the relative ratio of aglycones increases with fermentation [64].

Further, daidzein may be converted to *O*-desmethylangolensin and equol by gut microflora (Figure 2), although these metabolites are rarely found in fermented soy foods [65]. Similarly, genistein is metabolized to dihydrogenistein, 6′-hydroxy-*O*-desmethylangolensin, and 4-hydroxyphenyl-2-propionic acid by lactic acid bacteria and *Bifidobacteria* [66].

The recommended daily dose of soy isoflavones varies, and ranges from 40 to 120 mg according to different studies [67,68,69]. A recent study reported that the intake of 900 mg unconjugated soy isoflavones per day was safe and well tolerated in healthy postmenopausal women [67]. Significant increases in physiologically active isoflavone aglycone levels have been reported during fermentation processes, ranging from 16.74 µg/g soy when soaked to 31.44 µg/g soy at fermentation [68,69]. Furthermore, genistein levels in the fermented soybean products, *miso* and *natto*, ranged between 38.5 to 229.1 µg/g food and were higher than the soybean products, soy milk (1.9 to 13.9 µg/g food), and tofu (94.8 to 137.7 µg/g food), suggesting the β-glycosyl bonds of genistin were cleaved and transformed to genistein during fermentation [70].

**Figure 3:**
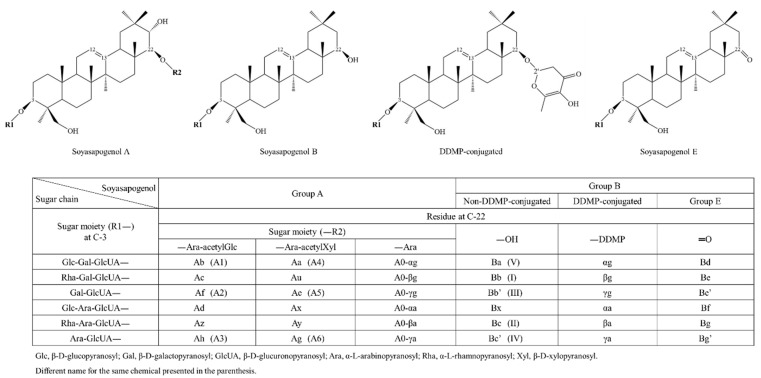
Chemical structure and nomenclature of soyasaponins (adapted from the literature with minor modifications [73,74]).

Soyasaponins, a group of distinctive compounds present in soybean, also undergo an extensive change during fermentation by fungi in the course of the *doenjang* manufacturing process. Moreover, 2,3-dihydro-2,5-dihydroxy-6-methyl-4H-pyran-4-one (DDMP)-conjugated soyasaponins are the molecular forms present in unprocessed soybeans, whereas unconjugated soyasaponins are mainly detected in processed soy products (Figure 3) [33]. The DDMP group in conjugated soyasaponins is easily removed from the parent compounds upon changes in temperature, pH, and solvent conditions [71]. After the fermentation of soybean using naturally occurring microorganisms, as in the preparation of *meju* and *doenjang*, the levels of unconjugated soyasaponins are increased, while the content of DDMP-conjugated soyasaponins is reduced by steaming [72]. The *meju* fermentation and brining steps have been reported to increase several unconjugated soyasaponins and decrease DDMP-conjugated soyasaponins. Fermentation of *meju* results in the conversion of most DDMP-conjugated soyasaponins to unconjugated soyasaponins I, III, and Be (Figure 3) [71].

During the *cheonggukjang* or *natto* manufacturing process, the fermentation of cooked soybean by bacteria, such as *Bacillus subtilis,* produces various metabolites, including peptones, peptides, amino acids, sugars, organic acids, natto kinase, levan, and polyglutamic acid, which is responsible for the sticky and slimy texture of these foods; moreover, these secondary metabolites dramatically affect the organoleptic and biological properties of the resultant products [75]. The contents of free amino acids and fatty acids were increased by protease and lipase activities, respectively, during *sufu* ripening period [21,76]. Eight biogenic amines, such as putrescine, cadaverine, spermidine, spermine, tyramine, 2-phenethylamine, histamine, and tryptamine, have been reported to be formed by the decarboxylation of free amino acids [76].

Maillard reaction products (MRPs), which are a group of well-known brownish compounds, are newly formed through chemical reaction between amino acids and sugars during the manufacturing process of fermented food products. Several MRPs, including fructose-lysine, were identified in soy sauce and miso [34]. MRPs have been reported to have antioxidant activity in vitro, cancer-preventive activity, and beneficial effects on gut health [77].

Many other compounds were also reported in different soy products. The anti-platelet alkaloids 1-methyl-1,2,3,4-tetrahydro-β-carboline and 1-methyl-β-carboline were detected in soy sauce. These two compounds suppress the maximal aggregation response induced by adenosine 5′-diphosphate, epinephrine, collagen, platelet-activating factor, and thrombin, respectively [34,78]. In addition, the asperparaline A, B, and C alkaloids were identified in the insoluble residue of whole soybean (called okara) fermented with *Aspergillus japonicus* JV-23, and were reported to have paralytic activity in silkworms [34,79]. Because these alkaloids are not present in raw soybean, the compounds were most likely to have been newly generated or introduced in the final product during the fermentation process.

*Tempeh*, which is a traditional Indonesian soy food made of fermented soybean, is popular because of its umami taste. Recently, a novel 15-amino-acid peptide (GENEEEDSGAIVTVK) that mainly contributes to the umami taste was identified [80].

In addition, soymilk contains low levels of water-soluble vitamins, such as riboflavin (vitamin B_2_) and cobalamin (vitamin B_12_). When it is fermented, the nutritional value of soymilk is enhanced by the high-level production of fat-soluble vitamin K_2_ (menaquinone-7) and water-soluble B vitamins, such as vitamins B_2_, B_6_, and B_12_ and folate [81,82].

Furanones, such as 4-hydroxy-2(or 5)-ethyl-5(or2)-methyl-3(2H)-furanone (HEMF), 4-hydroxy-2,5-dimethyl-3(2H)-furanone (HDMF), and 4-hydroxy-5-methyl-3(2H)-furanone (HMF), are probably formed from the Maillard reaction during yeast fermentation in the production of Japanese and Korean soy sauces [44,83,84]. HEMF is considered a key flavor compound in soy sauce, and HDMF and HMF are reported to have antioxidant activities and anti-carcinogenic effects [44,84].

## 4. Isoflavones and Neurodegenerative Diseases

AD and PD, the two most common neurodegenerative disorders, are characterized by a series of events encompassing abnormal protein aggregation, oxidative stress, neuroinflammation, and neuronal death. The canonical molecular changes of AD include the formation of insoluble amyloid beta peptide (Aβ) aggregates and neurofibrillary tangles (NFTs) primed by the hyperphosphorylated tau protein. PD is characterized by the intracellular accumulation of insoluble α-synuclein and the formation of Lewy bodies in neurons and glial cells [85]. This abnormal protein deposition contributes to neuronal dysfunction and degeneration, and further impairs the architecture and function of neural circuits in specific areas of the brain [85,86,87]. As oxidative stress and neuroinflammation are widely believed to be critical events in the pathological development of AD and PD, compounds with antioxidative and/or anti-inflammatory activity are expected to retard the progression of these two neurodegenerative diseases.

### 4.1. Isoflavones and AD

Soy isoflavones have been reported to have neuroprotective effects in various animal studies. In particular, the compounds were shown to attenuate AD-related pathology and reduce its progression. These effects of isoflavones are most likely associated with their antioxidative activity and their affinity for estrogen receptors [88].

In an experiment using a mouse model, soy isoflavones significantly attenuated galactose-induced oxidative stress, as evidenced by the reversal of the oxidative stress- and AD-related parameters, such as increased serum levels of thiobarbituric-acid-reactive substances in the brain and serum; increased levels of protein-bound carbonyls in the brain, kidney and liver; increased serum levels of advanced glycation end products; and increased expression of caspase-3 and Bax in splenocytes and of Aβ, β-amyloid precursor protein-cleaving enzyme-1 (BACE-1), and presenilin-1 (a subunit of γ-secretase) in the brain [89]. In addition, dietary isoflavones improved cognitive function in an ovariectomized rat model of AD [90].

Among the isoflavones, genistein was reported to ameliorate the Aβ-induced impairments responsible for neuronal death in AD animal models by exerting antioxidant activity, abating Aβ toxicity, inhibiting nitric oxide (NO) generation, and reducing tau pathology [90,91]. Another study also demonstrated that soy isoflavones reduced neuronal death and prevented degeneration of the nervous system through anti-inflammatory activity, regulation of cell signaling pathways, and antioxidant activity [92].

### 4.2. Isoflavones and PD

Genistein has been reported to protect dopaminergic neurons against lipopolysaccharide (LPS)-induced neuroinflammation in a PD model [93]. Moreover, it suppresses the production of superoxide, tumor necrosis factor alpha (TNFα), and nitric oxide (NO) in microglia and mesencephalic neuron–glia cultures [88]. Microglial cells in the brain are triggered by infection or injury, thereby releasing proinflammatory mediators, such as cytokines and reactive oxygen species (ROS) [94,95]. These cytokines and ROS may facilitate the formation of complexes with proteins, thus altering the function of crucial proteins and eventually causing cell death [95].

Interestingly, genistein was found to inhibit the accumulation and production of ROS and NO, thus protecting dopaminergic neurons from oxidative neuronal injury [88,94]. In addition, genistein exerted a protective effect on dopaminergic neurons in 1-methyl-4-phenyl-1,2,3,6-tetrahydropyridine (MPTP)-induced PD mice, which was likely attributable to the suppression of apoptotic neuronal cell death in midbrain via the upregulation of the *Bcl* 2 gene [96].

Daidzein was also reported to attenuate the LPS-induced expression of inflammatory mediators in a murine microglial BV-2 cell line. More specifically, pre-exposure of cells to daidzein significantly suppressed the expression of the proinflammatory factors NO and interleukin 6 (IL-6), with dampening of p38 mitogen-activated protein kinase (MAPK) phosphorylation, nuclear factor kappa-light-chain-enhancer of activated B cells (NF-κB) activation, and ROS production [97].

A recent report demonstrated that soy isoflavones attenuated the oxidative stress and inflammation induced by atrazine, as indicated by malondialdehyde accumulation and glutathione depletion, and increased TNFα and IL-6 release, respectively, in the substantia nigra. In addition, atrazine downregulated LC3-II and Beclin-1 and upregulated p62 in the substantial nigra, suggesting autophagy inhibition. In contrast, these effects were reversed by pre-treatment with soy isoflavones, suggesting that the compounds can restore the autophagy function of dopaminergic neurons in the substantia nigra. In fact, the dysregulation of autophagy is emerging as a major etiology of PD as reported by a number of studies [92,98,99,100]. In particular, restoring mitochondria-specific autophagy (termed mitophagy) in PD neurons has been demonstrated to prevent oxidative stress and dopaminergic neuronal damage in in vivo models and in patient-derived cells [98,101,102,103].

Furthermore, isoflavones (daidzein, genistein, biochanin A, and formononetin) induce mitochondrial biogenesis in myoblasts and renal cells through the activation of the NAD-dependent deacetylase sirtuin-1 (SIRT1)/peroxisome proliferator-activated receptor gamma coactivator 1-alpha (PGC-1α) pathway [104,105]. In turn, genistein upregulates the estrogen-related receptor alpha (ERR-α), ERR-β, PGC-1α, SIRT3, and the nuclear factor erythroid 2-related factor 2 (Nrf2) downstream enzymes, thus enhancing mitochondrial biogenesis and antioxidant responses [106].

Considering the effects of isoflavones on mitochondrial biogenesis and mitophagy in several tissues, it is highly plausible that isoflavones regulate mitochondrial homeostasis in the central nervous system (CNS) [107,108]. As several studies reported that PD is associated with dysregulated mitophagy, isoflavones in soy products offer a good therapeutic and/or preventive potential for PD [92,98,99,100].

## 5. Other Components in Fermented Soy Products and Neurodegenerative Diseases

As mentioned previously, it is most likely that antioxidants have a beneficial effect on neurodegenerative diseases, which are intimately related to oxidative stress. Fermented soy products have been reported to contain not only isoflavones, but also other antioxidant molecules.

### 5.1. Amino Acids and Peptides with Antioxidant Activity

Recent studies reported that the free amino acids, such as alanine, glycine, histidine, leucine, methionine, phenylalanine, tryptophan, tyrosine, and valine, present in peptides have antioxidant activity [109,110,111]. For instance, the radical scavenging activities of a peptide can be attributed to imidazole, indole, and phenol groups in histidine, tryptophan, and tyrosine, respectively [110,112], in which those chemical groups can easily donate protons to electron-deficient radicals [111].

Watanabe and coworkers also claimed that the amino acids and peptides formed during fermentation are responsible for antioxidant activity in the water-soluble fraction of *Rhizopus*-fermented *tempeh* [38]; the contents of free amino acids and peptides were found to increase during the aerobic fermentation with *Rhizopus*, with concomitant increase in antioxidant activity in the water-soluble fraction.

### 5.2. Soyasaponins

Soyasaponins have been reported to significantly inhibit NF-κB activation in LPS-treated microglial BV-2 cells. In particular, soyasapogenol B (SB) recovered LPS-induced cognitive deficit in a mouse model [113]. Furthermore, SB significantly increased cAMP response element-binding protein phosphorylation and brain-derived neurotrophic factor expression in LPS-treated mice and corticosterone-stimulated SH-SY5Y cells, and inhibited NF-κB activation in LPS-treated mice. Soyasaponin subclasses A1, A2, and I also inhibited the LPS-induced cyclooxygenase 2 (COX-2) expression in a dose-dependent manner through negative regulation of NF-kB. These studies consistently suggest that soyasaponins attenuate memory deficits by suppressing NF-κB-mediated inflammation [113,114].

## 6. Effect of Fermented Soy Products and Gut Microbiota on Neurodegenerative Diseases

It is well established that even the short-term dietary intake intake of fermented soy products can affect the composition of the human gut microbiota. For instance, an animal-based diet decreases the levels of Firmicutes, which metabolize dietary plant polysaccharides (*Eubacterium rectale*, *Roseburia*, and *Ruminococcus bromii*), while increasing the abundance of bile-tolerant microorganisms (*Alistipes*, *Bacteroides*, and *Bilophila*) [115,116]. A large-scale genome-wide analysis of human fecal samples demonstrated that the consumption of LAB-containing foods is reflected in the gut microbial balance [117].

### 6.1. Fermented Soy Products and Gut Microbiota

The fermentation of soybean confers unique sensory attribute, extends shelf life, modifies the nutritional quality and phytochemical profile, and enhances digestibility. In addition, the microorganisms used in fermentation themselves are a good source of prebiotics as well as probiotics.

Several recent studies have reported that the composition and structure of the gut microbiota could be changed by the consumption of fermented soy foods, such as fermented soymilk (yogurt), fermented tofu, soy paste (*doenjang*), and soy sauce (*ganjang*).

Fermented soymilk manufactured using *Lactobacillus* and *Bifidobacterium* affects populations of human fecal microbiota [118] in a desirable way, inducing effects that include alleviation of menopausal symptoms [119], control of hypercholesterolemia [120], modulation of mitogen-stimulated splenocyte proliferation, and TNFα production [121]. Fermented soy foods prepared with *Enterococci* and *Lactobacilli* were shown to increase these bacterial population in the gut. Similarly, water-soluble extracts of soybean fermented with *Lactobacillus helveticus* and *Enterococcus faecium* were reported to significantly increase the populations of *Enterococci*, *Lactobacilli*, and *Bifidobacteria* in the gut, and decrease the level of *Enterobacteriaceae*. The consumption of soymilk fermented by *Enterococcus faecium* or *Lactobacillus plantarum* significantly increased the populations of *Bifidobacterium*, *Enterococcus*, and *Lactobacillus* in the gut, while their effect on the abundance of gut *Clostridium* and *Bacteroides* was inconsistent. *Tempeh*, an Indonesian traditional fermented soy product, has been shown to increase the relative abundance of *Bifidobacterium*, *Lactobacillus*, *Escherichia coli*, and *Enterococcus* in an in vitro gut simulator model. In contrast, the consumption of *natto* was shown to increase the abundance of *Bacillus* and *Bifidobacterium* and decrease *Clostridia* and *Enterobacteriaceae* in the gut microbiota [122].

Fermented soy products manufactured by traditional methods in Korea are reported to contain high levels of *Bacillus* species, such as *Bacillus amyloliquefaciens*, *Bacillus subtilis*, and *Bacillus licheniformis* [123]. Nam and coworkers analyzed over 12,000 bacterial pyrosequences in a commercial brand of *cheonggukjang* and found that the vast majority of bacteria were assigned to the phylum Firmicutes (>95%), followed by Proteobacteria (<5%). Most of the Firmicutes were *Bacillus* species, although the levels of *Bacillus subtilis* (1.1–45.2%), *Bacillus licheniformis* (3.2–33.6%), and *Bacillus amyloliquefaciens* (0.2–9.2%) varied greatly according to brand. In some *cheonggukjang* samples, specific unclassified *Bacillus* species and lactic acid bacteria were the dominant microbes [124]. Kim and coworkers examined the bacterial communities in *meju* and also found that the predominant phylum was Firmicutes (93.6%) [125].

Recent studies demonstrated that the consumption of *cheonggukjang* fermented by *Bacillus subtilis* or *Bacillus amyloliquefaciens* increased the abundance of Bifidobacteriales and Lactobacillales in the gut. However, the population of Enterobacteriales, which are considered harmful bacteria, were lowered by a *cheonggukjang*-containing diet [126,127].

### 6.2. Gut Microbiota and Neurodegenerative Diseases

Emerging evidence strongly supports the notion that gut microbial composition and balance is closely associated with the risk of neurodegenerative diseases. The human gastrointestinal (GI) tract is estimated to harbor 100 trillion microorganisms, generally called the gut microbiota, which is determined by both host genetics and environmental factors [128,129]. An increasing number of studies have shown that the gut microbiota critically affects the function and development of the CNS [15].

#### 6.2.1. Gut Microbiota and PD

PD is a multifactorial neurodegenerative disease that is believed to be caused by both genetic changes and environmental factors. It is characterized by the deposition of toxic α-synuclein inclusions that lead to the death of dopaminergic neurons in the striatum and, consequently, motor dysfunction [130,131].

The pathogenesis of PD has been speculated to be associated with the GI tract as α-synuclein deposition was observed in the peripheral nervous system, especially in the enteric and pelvic plexus, of patients with PD [132]. A subsequent study suggested that the PD pathology originates from the peripheral organs in which α-synuclein is seeded, such as the GI tract and nasal cavity, before being retrograde transported to the cerebral cortex through the vagal nerve [132,133]. Furthermore, many patients with PD experience hyposmia and GI problems prior to the manifestation of classical PD symptoms, and patients with inflammatory bowel disease are also at a higher risk of developing PD [134]. Thus, the microbiota present in the GI tract are most likely involved in the pathogenesis of PD, in a direct or indirect manner.

The apoptotic death of dopaminergic neuronal cells in the substantia nigra has been widely believed to be triggered by oxidative stress [135,136]. Excessive production of ROS can cause oxidative damage in the brain of patients with PD, as shown by increased DNA damage and lipid peroxidation in the substantia nigra [18]. Increase in protein oxidation is also observed in many areas of the brain, with the substantia nigra being particularly susceptible [137,138]. Therefore, it is expected that antioxidants will attenuate and/or prevent the progression of PD. As mentioned above, naturally occurring antioxidants have a good potential to attenuate and/or prevent the progression of PD, which is associated with neuronal apoptosis triggered by excessive ROS production and a diminished capability to handle oxidative stress by dopaminergic neurons and/or neighboring tissues.

The fermentation of soybean produces several antioxidative compounds, such as peptides, aglycone forms of isoflavones, and soyasaponins; thus, it is most likely that fermented soy products alleviate the progression and aggravation of PD. Soy protein, which usually represents approximately 40% of the seed content, is degraded into peptides by microbial proteases during fermentation. The peptides produced from soy proteins exhibit various beneficial effects, including antioxidant activity, which regulate the redox balance in the gut and subsequently influence the gut microbiota in a positive manner [139,140].

Gut microbiota have been reported to preferentially ferment peptides over free amino acids [141], and some peptides possess high resistance against gastrointestinal digestion [142]; therefore, these peptides can affect the composition of gut microbiota and can be utilized by the gut microbiota to produce neurotransmitters, such as butyrate [141], which may improve the negative symptoms of neurodegenerative diseases [143]. In fact, butyrate greatly regulates immune functions and energy metabolism of hosts, and mediates host–microbe crosstalk through transporters (MCT1/SLC16A1; SMCT1/SLC5A8) and specific receptors (GPR43/FFAR2; GPR41/FFAR3; GPR109a/HCAR2). The effect of butyrate may also be mediated by the β-oxidation pathway and the inhibition of histone deacetylases (HDACs), leading to enhanced histone acetylation and gene expression in host cells. Butyrate is also widely used as an experimental pharmacological compound and, more recently, in neuroscience research [144,145]. Thus, this compound has been in the spotlight in research into the microbiota–gut–brain axis, to understand how gut-derived butyrate affects brain functions and behaviors, ranging from depression to neurodegenerative diseases and cognitive impairment [146].

Recent studies have demonstrated that a probiotic mixture of *Lactobacillus rhamnosus* GG, *Bifidobacterium animalis lactis*, and *Lactobacillus acidophilus* increases butyrate and subsequently rescues the nigral dopaminergic neurons from MPTP-and rotenone-induced neurotoxicity in a mouse model [147]. The neuroprotective effect of butyrate may be mediated by the upregulation of occludins, zonula occludens-1, and Bcl-2, and, in particular, the stimulation of the colonic glucagon-like peptide-1 (GLP-1) and the upregulation of brain GLP-1R [51].

#### 6.2.2. Gut Microbiota and AD

Disturbances in the composition of gut microbiota are related to immune activation and increased permeability of the gut barrier, thus leading to systemic inflammation, which, in turn, may compromise the blood–brain barrier and trigger neuroinflammation, neural damage, and neurodegeneration. More specifically, age-related alterations in the gut microbiota characterized by lowered diversity and stability may lead to an incessant inflammatory state of the gut mucosa, ultimately resulting in chronic systemic inflammation, including neuroinflammation [148,149,150].

It has been reported that the gut microbial composition of patients with AD is hallmarked by a decreased abundance of Firmicutes and Actinobacteria, and an increased abundance of Bacteroidetes and Proteobacteria. More specifically, the families that were reduced within the Firmicutes phylum include Clostridiaceae, Mogibacteriaceae, Peptostreptococcaceae, Ruminococcaceae, and Turicibacteraceae. The Acinetobacteria and Bifidobacteriaceae families were reduced in the gut of patients with AD. In contrast, Bacteroidaceae and Rikenellaceae within the Bacteroidetes phylum were increased in these individuals. In general, patients with AD harbor an increased number of proinflammatory bacteria, such as Bacteroidetes and Proteobacteria (*Escherichia* and *Shigella*), and have decreased anti-inflammatory bacteria (Firmicutes, *Bifidobacterium*, and *Eubacterium rectale*). However, additional research is required to establish a solid correlation between gut microbiota and AD, as the alterations in the gut microbiota of patients with AD were not consistent among studies [151].

A plant-based salutary foods diet containing probiotics, soybeans, nuts, omega-3 polyunsaturated fatty acids, and antioxidants, as well as a low intake of saturated fats, animal-derived foods, and refined sugar, has been reported to inhibit the inflammatory response, attenuate insulin resistance, and lower the risk of cognitive impairment and AD [152,153].

The intake of *cheonggukjang* fermented with *Bacillus* species prevents and alleviates the memory impairment observed in patients with AD and cerebral ischemic condition. In particular, *cheonggukjang*, which contains a high poly-L-γ-glutamic acid (γ-PGA), exhibited better efficacy for improving glucose metabolism and neuronal cell survival than did a low level of γ-PGA [126], although the neuroprotective effect and related mechanism(s) of γ-PGA remain unclear.

Yang and colleagues reported that soybeans fermented with *Bacillus licheniformis* enhanced cognitive function in diabetic rats with AD-type dementia [154]. Several proteinases produced by *Bacillus pumilus* and *Bacillus subtilis* and present in fermented soy products possess amyloid-degrading activity; therefore, they can be developed into anti-aggregation drugs [155,156], although many hurdles in the delivery of the proteases to target sites are anticipated. Another study found that *Bacillus subtilis,* a microorganism that is predominant in traditionally made *cheonggukjang,* restored the lifespan of *Caenorhabditis elegans* strains that expressing Aβ to values similar to the life expectancy of the wild-type strain [157]. The direct effects of microorganism in AD models are believed to be associated with the ability of *Bacillus subtilis* to biosynthesize quorum-sensing peptides (i.e., the competence and sporulation factor) and form a gut-associated biofilm, which is associated with the anti-aging effect.

## 7. Conclusions

The accumulation of toxic unique proteins or peptides characterized by abnormal conformational properties inside neuronal cells in the brain is a common feature of AD and PD, which are the two most prevalent neurodegenerative diseases with an incidence that keeps increasing globally. These peptides or proteins usually exert deleterious effects on the CNS through the generation of ROS, exacerbation of inflammation, alteration of mitochondrial homeostasis, and their combinations.

**Figure 4:**
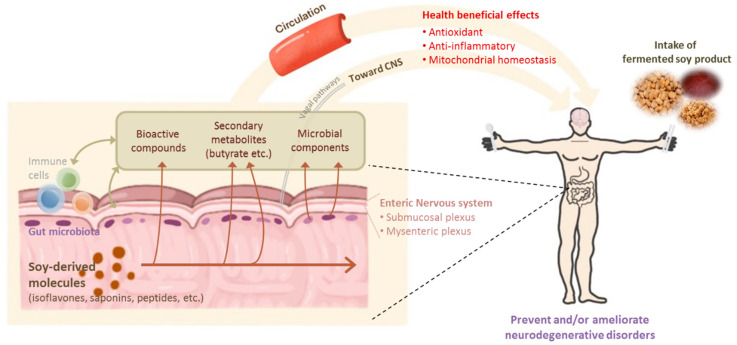
Health-beneficial effects of fermented soy products on neurodegenerative disorders.

Fermented soybean products have well-known beneficial effects on neurodegenerative diseases and afford a variety of health benefits, such as the prevention of several chronic diseases. In particular, the free isoflavones generated during the fermentation of cooked soybean may attenuate the progression of AD and PD via antioxidant activity and the restoration of ROS-mediated mitochondrial dysfunction, as illustrated in Figure 4.

Recent studies also suggested that the regulation of the gut microbiome by fermented soy products can modulate neurodegenerative diseases through metabolites produced by microbial fermentation, such as butyrate, or by changing the gut microbial composition in a beneficial fashion.

However, clinical data regarding the therapeutic or preventive effects of fermented soybean products in neurodegenerative diseases are limited. Further research using large, long-term clinical trials to evaluate fermented soybean products and their components would be helpful in making specific dietary recommendations to patients with AD and PD.

## Figures and Tables

**Table 1:** Fermented soy products and phytochemicals.

Fermented Soy Products	Phytochemicals	Biological Functions	References
*Cheonggukjang (natto)*	Free isoflavones, levan, γPGA, natto kinase, vitamin K	Antioxidant, anti-hypertension, fibrinolysis, bone health	[29,30,31]
*Doenjang (miso)*	Free isoflavones, non-DDMP-conjugated soyasaponins (I, III, Be), peptides, amino acids, MRPs, kojic acid	Antioxidant, anti-obesity, anti-tumorigenic, anti-hypertension, anti-sarcopenia, skin whitening, immune modulation, sympathetic nerve activity, anti-diabetic activity	[32,33,34,35,36,37,38,39,40,41,42,43]
*Ganjang*	Amino acids, peptides, MRPs, 1-methyl-1,2,3,4-tetrahydro-β-carboline and 1-methyl-β-carboline	Anti-platelet activity, anti-allergenicity, anti-hypertension	[34,44,45,46,47]
*Douchi*	Subtilisin DFE, isoflavones, peptides	Antioxidant, fibrinolysis, α-amylase inhibition, ACE inhibition, anti-acetylcholine esterase	[20,48,49,50,51]
*Tempeh*	6,7,4’-trihydroxyisoflavone, isoflavones, peptides	Antioxidant, cognitive improvement, BACE1 inhibition,	[52,53,54]
*Gochujang*	Capsaicin, free isoflavones	Antioxidant, anti-obesity	[55,56,57]
*Fermented soymilk*	Free isoflavones, water-soluble vitamins (B_2_, B_6_, folate, and B_12_), vitamin K_2_ (menaquinone-7)	Antioxidant, anti-obesity, increased mineral bioavailability, anti-mutagenic, hypocholesterolemic effects	[58,59,60,61,62,63]

γPGA, gamma-polyglutamate; MRPs, Maillard reaction products, DDMP, 2,3-Dihydro-2,5-dihydroxy-6-methyl-4H-pyran-4-one; ACE, angiotensin-converting enzyme.

## References

[1] United Nations Department of Economic and Social Affairs Population Division (2019). World Population Prospects 2019: Highlights. ST/ESA/SER.A/423. https://population.un.org/wpp/Publications/Files/WPP2019_Highlights.pdf..

[2] (2019). Selected Results of the 2019 UN World Population Projections. Popul. Dev. Rev..

[3] Mariani E., Polidori M.C., Cherubini A., Mecocci P. (2005). Oxidative stress in brain aging, neurodegenerative and vascular diseases: An overview. J. Chromatogr. B Analyt. Technol. Biomed. Life Sci..

[4] Fifel K., Videnovic A. (2020). Circadian alterations in patients with neurodegenerative diseases: Neuropathological basis of underlying network mechanisms. Neurobiol. Dis..

[5] Canter R.G., Penney J., Tsai L.H. (2016). The road to restoring neural circuits for the treatment of Alzheimer’s disease. Nature.

[6] Ahmad A., Hayat I., Arif S., Masud T., Khalid N., Ahmed A. (2014). Mechanisms Involved in the Therapeutic Effects of Soybean (Glycine Max). Int. J. Food Prop..

[7] Isanga J., Zhang G.N. (2008). Soybean bioactive components and their implications to health—A review. Food Rev. Int..

[8] Wang Q., Ge X., Tian X., Zhang Y., Zhang J., Zhang P. (2013). Soy isoflavone: The multipurpose phytochemical (Review). Biomed. Rep..

[9] Ko J.W., Chung Y.S., Kwak C.S., Kwon Y.H. (2019). Doenjang, a Korean Traditional Fermented Soybean Paste, Ameliorates Neuroinflammation and Neurodegeneration in Mice Fed a High-Fat Diet. Nutrients.

[10] Jayachandran M., Xu B.J. (2019). An insight into the health benefits of fermented soy products. Food Chem..

[11] Cao Z.H., Green-Johnson J.M., Buckley N.D., Lin Q.Y. (2019). Bioactivity of soy-based fermented foods: A review. Biotechnol. Adv..

[12] Kim B., Hong V.M., Yang J., Hyun H., Im J.J., Hwang J., Yoon S., Kim J.E. (2016). A Review of Fermented Foods with Beneficial Effects on Brain and Cognitive Function. Prev. Nutr. Food Sci..

[13] Kim D.C., Quang T.H., Yoon C.S., Ngan N.T.T., Lim S.I., Lee S.Y., Kim Y.C., Oh H. (2016). Anti-neuroinflammatory activities of indole alkaloids from kanjang (Korean fermented soy source) in lipopolysaccharide-induced BV2 microglial cells. Food Chem..

[14] Katagiri R., Sawada N., Goto A., Yamaji T., Iwasaki M., Noda M., Iso H., Tsugane S., Japan Public Health Center-based Prospective Study Group (2020). Association of soy and fermented soy product intake with total and cause specific mortality: Prospective cohort study. BMJ.

[15] Fung T.C., Olson C.A., Hsiao E.Y. (2017). Interactions between the microbiota, immune and nervous systems in health and disease. Nat. Neurosci..

[16] Sanchez B., Delgado S., Blanco-Miguez A., Lourenco A., Gueimonde M., Margolles A. (2017). Probiotics, gut microbiota, and their influence on host health and disease. Mol. Nutr. Food Res..

[17] Lee H.J., Hwang Y.H., Kim D.H. (2018). Lactobacillus plantarum C29-Fermented Soybean (DW2009) Alleviates Memory Impairment in 5XFAD Transgenic Mice by Regulating Microglia Activation and Gut Microbiota Composition. Mol. Nutr. Food Res..

[18] Shin D., Jeong D. (2015). Korean traditional fermented soybean products: Jang. J. Ethn. Foods.

[19] Lee Y.J., Kim N.Y., Kim U.S., Han M.J. (2017). Development of Lentil Cheonggukjang Fermented by Bacillus subtilis Isolated from Traditional Soy Sauce. J. Korean Soc. Food Cult..

[20] Wang D., Wang L.J., Zhu F.X., Zhu J.Y., Chen X.D., Zou L., Saito M., Li L.T. (2008). In vitro and in vivo studies on the antioxidant activities of the aqueous extracts of Douchi (a traditional Chinese salt-fermented soybean food). Food Chem..

[21] Li Y.Y., Yu R.C., Chou C.C. (2010). Some biochemical and physical changes during the preparation of the enzyme-ripening sufu, a fermented product of soybean curd. J. Agric. Food Chem..

[22] Han B.-Z., Rombouts F.M., Nout M.J.R. (2001). A Chinese fermented soybean food. Int. J. Food Microbiol..

[23] Licandro H., Ho P.H., Nguyen T.K.C., Petchkongkaew A., Nguyen H.V., Chu-Ky S., Nguyen T.V.A., Lorn D., Wache Y. (2020). How fermentation by lactic acid bacteria can address safety issues in legumes food products?. Food Control.

[24] Anderson R.L., Wolf W.J. (1995). Compositional Changes in Trypsin-Inhibitors, Phytic Acid, Saponins and Isoflavones Related to Soybean Processing. J. Nutr..

[25] Sindhu S.C., Khetarpaul N. (2001). Probiotic fermentation of indigenous food mixture: Effect on antinutrients and digestibility of starch and protein. J. Food Compost. Anal..

[26] Osawa R., Kuroiso K., Goto S., Shimizu A. (2000). Isolation of tannin-degrading lactobacilli from humans and fermented foods. Appl. Environ. Microbiol..

[27] Rodriguez H., Curiel J.A., Landete J.M., de las Rivas B., de Felipe F.L., Gomez-Cordoves C., Mancheno J.M., Munoz R. (2009). Food phenolics and lactic acid bacteria. Int. J. Food Microbiol..

[28] Cuadrado C., Hajos G., Burbano C., Pedrosa M.M., Ayet G., Muzquiz M., Pusztai A., Gelencser E. (2002). Effect of natural fermentation on the lectin of lentils measured by immunological methods. Food Agric. Immunol..

[29] Sumi H., Hamada H., Tsushima H., Mihara H., Muraki H. (1987). A novel fibrinolytic enzyme (nattokinase) in the vegetable cheese Natto; a typical and popular soybean food in the Japanese diet. Experientia.

[30] Ikeda Y., Iki M., Morita A., Kajita E., Kagamimori S., Kagawa Y., Yoneshima H. (2006). Intake of fermented soybeans, natto, is associated with reduced bone loss in postmenopausal women: Japanese population-based osteoporosis (JPOS) study. J. Nutr..

[31] Ogawa Y., Yamaguchi F., Yuasa K., Tahara Y. (1997). Efficient production of gamma-polyglutamic acid by Bacillus subtilis (natto) in jar fermenters. Biosci. Biotechnol. Biochem..

[32] da Silva L.H., Celeghini R.M.S., Chang Y.K. (2011). Effect of the fermentation of whole soybean flour on the conversion of isoflavones from glycosides to aglycones. Food Chem..

[33] Hu J., Lee S.O., Hendrich S., Murphy P.A. (2002). Quantification of the group B soyasaponins by high-performance liquid chromatography. J. Agric. Food Chem..

[34] Kang J., Badger T.M., Ronis M.J., Wu X. (2010). Non-isoflavone phytochemicals in soy and their health effects. J. Agric. Food Chem..

[35] Coward L., Barnes N.C., Setchell K.D.R., Barnes S. (1993). Genistein, Daidzein, and Their Beta-Glycoside Conjugates—Antitumor Isoflavones in Soybean Foods from American and Asian Diets. J. Agric. Food Chem..

[36] Okouchi R., Sakanoi Y., Tsuduki T. (2019). Miso (Fermented Soybean Paste) Suppresses Visceral Fat Accumulation in Mice, Especially in Combination with Exercise. Nutrients.

[37] Baggott J.E., Ha T., Vaughn W.H., Juliana M.M., Hardin J.M., Grubbs C.J. (1990). Effect of miso (Japanese soybean paste) and NaCl on DMBA-induced rat mammary tumors. Nutr. Cancer.

[38] Watanabe N., Fujimoto K., Aoki H. (2007). Antioxidant activities of the water-soluble fraction in tempeh-like fermented soybean (GABA-tempeh). Int. J. Food Sci. Nutr..

[39] Takahashi F., Hashimoto Y., Kaji A., Sakai R., Kawate Y., Okamura T., Kitagawa N., Okada H., Nakanishi N., Majima S. (2020). Habitual Miso (Fermented Soybean Paste) Consumption Is Associated with a Low Prevalence of Sarcopenia in Patients with Type 2 Diabetes: A Cross-Sectional Study. Nutrients.

[40] Bentley R. (2006). From miso, sake and shoyu to cosmetics: A century of science for kojic acid. Nat. Prod. Rep..

[41] Kumazawa T., Nishimura A., Asai N., Adachi T. (2018). Isolation of immune-regulatory Tetragenococcus halophilus from miso. PLoS ONE.

[42] Ito K. (2020). Review of the health benefits of habitual consumption of miso soup: Focus on the effects on sympathetic nerve activity, blood pressure, and heart rate. Environ. Health Prev. Med..

[43] Kwon D.Y., Daily J.W., Kim H.J., Park S. (2010). Antidiabetic effects of fermented soybean products on type 2 diabetes. Nutr. Res..

[44] Kataoka S. (2005). Functional effects of Japanese style fermented soy sauce (shoyu) and its components. J. Biosci. Bioeng..

[45] Kobayashi M. (2005). Immunological functions of soy sauce: Hypoallergenicity and antiallergic activity of soy sauce. J. Biosci. Bioeng..

[46] Nakahara T., Sano A., Yamaguchi H., Sugimoto K.R.I., Chikata H., Kinoshita E., Uchida R. (2010). Antihypertensive Effect of Peptide-Enriched Soy Sauce-Like Seasoning and Identification of Its Angiotensin I-Converting Enzyme Inhibitory Substances. J. Agric. Food Chem..

[47] Li Y., Zhao M., Parkin K.L. (2011). beta-carboline derivatives and diphenols from soy sauce are in vitro quinone reductase (QR) inducers. J. Agric. Food Chem..

[48] Peng Y., Huang Q., Zhang R.H., Zhang Y.Z. (2003). Purification and characterization of a fibrinolytic enzyme produced by Bacillus amyloliquefaciens DC-4 screened from douchi, a traditional Chinese soybean food. Comp. Biochem. Physiol. B Biochem. Mol. Biol..

[49] Chen J., Cheng Y.Q., Yamaki K., Li L.T. (2007). Anti-alpha-glucosidase activity of Chinese traditionally fermented soybean (douchi). Food Chem..

[50] Zhang J.H., Tatsumi E., Ding C.H., Li L.T. (2006). Angiotensin I-converting enzyme inhibitory peptides in douchi, a Chinese traditional fermented soybean product. Food Chem..

[51] Liu J.M., Wang F.Y., Liu S.Z., Du J.M., Hu X.Z., Xiong J.J., Fang R.C., Chen W.Q., Sun J. (2017). Sodium butyrate exerts protective effect against Parkinson’s disease in mice via stimulation of glucagon like peptide-1. J. Neurol. Sci..

[52] Esaki H., Onozaki H., Kawakishi S., Osawa T. (1996). New antioxidant isolated from tempeh. J. Agric. Food Chem..

[53] Handajani Y.S., Turana Y., Yogiara Y., Widjaja N.T., Sani T.P., Christianto G.A.M., Suwanto A. (2020). Tempeh Consumption and Cognitive Improvement in Mild Cognitive Impairment. Dement. Geriatr. Cogn. Disord..

[54] Ahmad A., Ramasamy K., Majeed A.A., Mani V. (2015). Enhancement of beta-secretase inhibition and antioxidant activities of tempeh, a fermented soybean cake through enrichment of bioactive aglycones. Pharm. Biol..

[55] Shin H.W., Jang E.S., Moon B.S., Lee J.J., Lee D.E., Lee C.H., Shin C.S. (2016). Anti-obesity effects of gochujang products prepared using rice koji and soybean meju in rats. J. Food Sci. Tech. Mys..

[56] Yang H.J., Lee Y.S., Choi I.S. (2018). Comparison of physicochemical properties and antioxidant activities of fermented soybean-based red pepper paste, Gochujang, prepared with five different red pepper (*Capsicum annuum* L.) varieties. J. Food Sci. Tech. Mys..

[57] Son H.K., Shin H.W., Jang E.S., Moon B.S., Lee C.H., Lee J.J. (2020). Gochujang prepared using rice and wheat koji partially alleviates high-fat diet-induced obesity in rats. Food Sci. Nutr..

[58] Wang Y.C., Yu R.C., Chou C.C. (2006). Antioxidative activities of soymilk fermented with lactic acid bacteria and bifidobacteria. Food Microbiol..

[59] Zhu Y.Y., Thakur K., Feng J.Y., Cai J.S., Zhang J.G., Hu F., Russo P., Spano G., Wei Z.J. (2020). Riboflavin-overproducing lactobacilli for the enrichment of fermented soymilk: Insights into improved nutritional and functional attributes. Appl. Microbiol. Biotechnol..

[60] Zhang X.L., Wu Y.F., Wang Y.S., Wang X.Z., Piao C.H., Liu J.M., Liu Y.L., Wang Y.H. (2017). The protective effects of probiotic-fermented soymilk on high-fat diet-induced hyperlipidemia and liver injury. J. Funct. Foods.

[61] Rekha C.R., Vijayalakshmi G. (2010). Bioconversion of isoflavone glycosides to aglycones, mineral bioavailability and vitamin B complex in fermented soymilk by probiotic bacteria and yeast. J. Appl. Microbiol..

[62] Hsieh M.L., Chou C.C. (2006). Mutagenicity and antimutagenic effect of soymilk fermented with lactic acid bacteria and bifidobacteria. Int. J. Food Microbiol..

[63] Kobayashi M., Hirahata R., Egusa S., Fukuda M. (2012). Hypocholesterolemic Effects of Lactic Acid-Fermented Soymilk on Rats Fed a High Cholesterol Diet. Nutrients.

[64] Nam D.H., Kim H.J., Lim J.S., Kim K.H., Park C.S., Kim J.H., Lim J., Kwon D.Y., Kim I.H., Kim J.S. (2011). Simultaneous Enhancement of Free Isoflavone Content and Antioxidant Potential of Soybean by Fermentation with Aspergillus oryzae. J. Food Sci..

[65] Chen Y.M., Shih T.W., Chiu C.P., Pan T.M., Tsai T.Y. (2013). Effects of lactic acid bacteria-fermented soy milk on melanogenesis in B16F0 melanocytes. J. Funct. Foods.

[66] Peiroten A., Gaya P., Alvarez I., Landete J.M. (2020). Production of O-desmethylangolensin, tetrahydrodaidzein, 6′-hydroxy-*O*-desmethylangolensin and 2-(4-hydroxyphenyl)-propionic acid in fermented soy beverage by lactic acid bacteria and Bifidobacterium strains. Food Chem..

[67] Pop E.A., Fischer L.M., Coan A.D., Gitzinger M., Nakamura J., Zeisel S.H. (2008). Effects of a high daily dose of soy isoflavones on DNA damage, apoptosis, and estrogenic outcomes in healthy postmenopausal women: A phase I clinical trial. Menopause.

[68] Chang T.S., Ding H.Y., Tai S.S., Wu C.Y. (2007). Metabolism of the soy isoflavones daidzein and genistein by fungi used in the preparation of various fermented soybean foods. Biosci. Biotechnol. Biochem..

[69] Piao Y.Z., Eun J.B. (2020). Physicochemical characteristics and isoflavones content during manufacture of short-time fermented soybean product (cheonggukjang). J. Food Sci. Technol..

[70] Fukutake M., Takahashi M., Ishida K., Kawamura H., Sugimura T., Wakabayashi K. (1996). Quantification of Genistein and Genistin in Soybeans and Soybean Products. Food Chem. Toxicol..

[71] Zhang W., Tang F.Y., Yeo M.C., Popovich D.G. (2012). Fermentation of Group B Soyasaponins with Probiotic Lactobacillus Rhamnosus. J. Food Biochem..

[72] Lee S.Y., Lee S., Lee S., Oh J.Y., Jeon E.J., Ryu H.S., Lee C.H. (2014). Primary and secondary metabolite profiling of doenjang, a fermented soybean paste during industrial processing. Food Chem..

[73] Zhang W., Popovich D.G. (2009). Chemical and Biological Characterization of Oleanane Triterpenoids from Soy. Molecules.

[74] Krishnamurthy P., Tsukamoto C., Takahashi Y., Hongo Y., Singh R.J., Lee J.D., Chung G. (2014). Comparison of saponin composition and content in wild soybean (Glycine soja Sieb. and Zucc.) before and after germination. Biosci. Biotechnol. Biochem..

[75] Jeon H.L., Yang S.J., Son S.H., Kim W.S., Lee N.K., Paik H.D. (2018). Evaluation of probiotic Bacillus subtilis P229 isolated from cheonggukjang and its application in soybean fermentation. Lebensm. Wiss. Technol..

[76] Guan R.-F., Liu Z.-F., Zhang J.-J., Wei Y.-X., Wahab S., Liu D.-H., Ye X.-Q. (2013). Investigation of biogenic amines in sufu (furu): A Chinese traditional fermented soybean food product. Food Control.

[77] Somoza V. (2005). Five years of research on health risks and benefits of Maillard reaction products: An update. Mol. Nutr. Food Res..

[78] Tsuchiya H., Sato M., Watanabe I. (1999). Antiplatelet activity of soy sauce as functional seasoning. J. Agric. Food Chem..

[79] Hayashi H., Nishimoto Y., Akiyama K., Nozaki H. (2000). New paralytic alkaloids, asperparalines A, B and C, from Aspergillus japonicus JV-23. Biosci. Biotechnol. Biochem..

[80] Amin M.N.G., Kusnadi J., Hsu J.L., Doerksen R.J., Huang T.C. (2020). Identification of a novel umami peptide in tempeh (Indonesian fermented and its mechanism to the umami T1R. Food Chem..

[81] Kamao M., Suhara Y., Tsugawa N., Uwano M., Yamaguchi N., Uenish K., Ishida H., Sasaki S., Okano T. (2007). Vitamin K content of foods and dietary vitamin K intake in Japanese young women. J. Nutr. Sci. Vitaminol..

[82] Zhu Y.-Y., Thakur K., Feng J.-Y., Cai J.-S., Zhang J.-G., Hu F., Wei Z.-J. (2020). B-vitamin enriched fermented soymilk: A novel strategy for soy-based functional foods development. Trends Food Sci. Technol..

[83] Zhang Y.F., Tao W.Y. (2009). Flavor and taste compounds analysis in Chinese solid fermented soy sauce. Afr. J. Biotechnol..

[84] Kataoka S., Liu W., Albright K., Storkson J., Pariza M. (1997). Inhibition of Benzo[a]pyrene-induced Mouse Fore, stomach Neoplasia and Reduction of H202 Concentration in Human Polymorphonuclear Leucocytes by Flavour Components of Japanese-style Fermented Soy Sauce. Food Chem. Toxicol..

[85] Spires-Jones T.L., Attems J., Thal D.R. (2017). Interactions of pathological proteins in neurodegenerative diseases. Acta Neuropathol..

[86] Dugger B.N., Dickson D.W. (2017). Pathology of Neurodegenerative Diseases. Cold Spring Harb. Perspect. Biol..

[87] Xie A.M., Gao J., Xu L., Meng D.M. (2014). Shared Mechanisms of Neurodegeneration in Alzheimer’s Disease and Parkinson’s Disease. Biomed Res. Int..

[88] Soni M., Rahardjo T.B., Soekardi R., Sulistyowati Y., Lestariningsih, Yesufu-Udechuku A., Irsan A., Hogervorst E. (2014). Phytoestrogens and cognitive function: A review. Maturitas.

[89] Hsieh H.M., Wu W.M., Hu M.L. (2009). Soy isoflavones attenuate oxidative stress and improve parameters related to aging and Alzheimer’s disease in C57BL/6J mice treated with D-galactose. Food Chem. Toxicol..

[90] Sarkaki A., Amani R., Badavi M., Moghaddam A.Z., Aligholi H., Safahani M., Haghighizadeh M.H. (2008). Pre-treatment effect of different doses of soy isoflavones on spatial learning and memory in an ovariectomized animal model of Alzheimer’s disease. Pak. J. Biol. Sci..

[91] Uddin M.S., Kabir M.T. (2019). Emerging Signal Regulating Potential of Genistein Against Alzheimer’s Disease: A Promising Molecule of Interest. Front. Cell Dev. Biol..

[92] Lu Y., An Y., Lv C., Ma W., Xi Y., Xiao R. (2018). Dietary soybean isoflavones in Alzheimer’s disease prevention. Asia Pac. J. Clin. Nutr..

[93] Wang X.J., Chen S.D., Ma G.Z., Ye M., Lu G.Q. (2005). Genistein protects dopaminergic neurons by inhibiting microglial activation. Neuroreport.

[94] Gao H.M., Jiang J., Wilson B., Zhang W., Hong J.S., Liu B. (2002). Microglial activation-mediated delayed and progressive degeneration of rat nigral dopaminergic neurons: Relevance to Parkinson’s disease. J. Neurochem..

[95] Hussain G., Zhang L.B., Rasul A., Anwar H., Sohail M.U., Razzaq A., Aziz N., Shabbir A., Ali M., Sun T. (2018). Role of Plant-Derived Flavonoids and Their Mechanism in Attenuation of Alzheimer’s and Parkinson’s Diseases: An Update of Recent Data. Molecules.

[96] Liu L.X., Chen W.F., Xie J.X., Wong M.S. (2008). Neuroprotective effects of genistein on dopaminergic neurons in the mice model of Parkinson’s disease. Neurosci. Res..

[97] Chinta S.J., Ganesan A., Reis-Rodrigues P., Lithgow G.J., Andersen J.K. (2013). Anti-Inflammatory Role of the Isoflavone Diadzein in Lipopolysaccharide-Stimulated Microglia: Implications for Parkinson’s Disease. Neurotox. Res..

[98] Wang Y., Liu N., Lu B.W. (2019). Mechanisms and roles of mitophagy in neurodegenerative diseases. CNS Neurosci. Ther..

[99] Killackey S.A., Philpott D.J., Girardin S.E. (2020). Mitophagy pathways in health and disease. J. Cell Biol..

[100] Montava-Garriga L., Ganley I.G. (2020). Outstanding Questions in Mitophagy: What We Do and Do Not Know. J. Mol. Biol..

[101] Scorziello A., Borzacchiello D., Sisalli M.J., Di Martino R., Morelli M., Feliciello A. (2020). Mitochondrial Homeostasis and Signaling in Parkinson’s Disease. Front. Aging Neurosci..

[102] Martinez-Vicente M. (2017). Neuronal Mitophagy in Neurodegenerative Diseases. Front. Mol. Neurosci..

[103] Yamano K., Matsuda N., Tanaka K. (2016). The ubiquitin signal and autophagy: An orchestrated dance leading to mitochondrial degradation. EMBO Rep..

[104] Apelt J., Schliebs R. (2001). beta-Amyloid-induced glial expression of both pro- and anti-inflammatory cytokines in cerebral cortex of aged transgenic Tg2576 mice with Alzheimer plaque pathology. Brain Res..

[105] Valles S.L., Dolz-Gaiton P., Gambini J., Borras C., Lloret A., Pallardo F.V., Vina J. (2010). Estradiol or genistein prevent Alzheimer’s disease-associated inflammation correlating with an increase PPAR gamma expression in cultured astrocytes. Brain Res..

[106] Zhou X., Yuan L., Zhao X., Hou C., Ma W., Yu H., Xiao R. (2014). Genistein antagonizes inflammatory damage induced by beta-amyloid peptide in microglia through TLR4 and NF-kappaB. Nutrition.

[107] Davinelli S., De Stefani D., De Vivo I., Scapagnini G. (2020). Polyphenols as Caloric Restriction Mimetics Regulating Mitochondrial Biogenesis and Mitophagy. Trends Endocrinol. Metab..

[108] Yessenkyzy A., Saliev T., Zhanaliyeva M., Masoud A.R., Umbayev B., Sergazy S., Krivykh E., Gulyayev A., Nurgozhin T. (2020). Polyphenols as Caloric-Restriction Mimetics and Autophagy Inducers in Aging Research. Nutrients.

[109] Ajibola C.F., Fashakin J.B., Fagbemi T.N., Aluko R.E. (2011). Effect of Peptide Size on Antioxidant Properties of African Yam Bean Seed (*Sphenostylis stenocarpa*) Protein Hydrolysate Fractions. Int. J. Mol. Sci..

[110] Guo H., Kouzuma Y., Yonekura M. (2009). Structures and properties of antioxidative peptides derived from royal jelly protein. Food Chem..

[111] Sanjukta S., Rai A.K. (2016). Production of bioactive peptides during soybean fermentation and their potential health benefits. Trends Food Sci. Technol..

[112] Nam K.A., You S.G., Kim S.M. (2008). Molecular and physical characteristics of squid (*Todarodes pacificus*) skin collagens and biological properties of their enzymatic hydrolysates. J. Food Sci..

[113] Lee H.J., Lim S.M., Ko D.B., Jeong J.J., Hwang Y.H., Kim D.H. (2017). Soyasapogenol B and Genistein Attenuate Lipopolysaccharide-Induced Memory Impairment in Mice by the Modulation of NF-kappaB-Mediated BDNF Expression. J. Agric. Food Chem..

[114] Zha L.Y., Chen J.D., Sun S.X., Mao L.M., Chu X.W., Deng H., Cai J.W., Li X.F., Liu Z.Q., Cao W.H. (2014). Soyasaponins Can Blunt Inflammation by Inhibiting the Reactive Oxygen Species-Mediated Activation of PI3K/Akt/NF-kB Pathway. PLoS ONE.

[115] David L.A., Maurice C.F., Carmody R.N., Gootenberg D.B., Button J.E., Wolfe B.E., Ling A.V., Devlin A.S., Varma Y., Fischbach M.A. (2014). Diet rapidly and reproducibly alters the human gut microbiome. Nature.

[116] Muegge B.D., Kuczynski J., Knights D., Clemente J.C., Gonzalez A., Fontana L., Henrissat B., Knight R., Gordon J.I. (2011). Diet Drives Convergence in Gut Microbiome Functions Across Mammalian Phylogeny and Within Humans. Science.

[117] Pasolli E., De Filippis F., Mauriello I.E., Cumbo F., Walsh A.M., Leech J., Cotter P.D., Segata N., Ercolini D. (2020). Large-scale genome-wide analysis links lactic acid bacteria from food with the gut microbiome. Nat. Commun..

[118] Cheng I.C., Shang H.F., Lin T.F., Wang T.H., Lin H.S., Lin S.H. (2005). Effect of fermented soy milk on the intestinal bacterial ecosystem. World J. Gastroenterol..

[119] Chiang S.S., Pan T.M. (2011). Antiosteoporotic Effects of Lactobacillus-Fermented Soy Skim Milk on Bone Mineral Density and the Microstructure of Femoral Bone in Ovariectomized Mice. J. Agric. Food Chem..

[120] Cavallini D.C.U., Manzoni M.S.J., Bedani R., Roselino M.N., Celiberto L.S., Vendramini R.C., de Valdez G.F., Abdalla D.S.P., Pinto R.A., Rosetto D. (2016). Probiotic Soy Product Supplemented with Isoflavones Improves the Lipid Profile of Moderately Hypercholesterolemic Men: A Randomized Controlled Trial. Nutrients.

[121] Appukutty M., Ramasamy K., Rajan S., Vellasamy S., Ramasamy R., Radhakrishnan A.K. (2015). Effect of orally administered soy milk fermented with Lactobacillus plantarum LAB12 and physical exercise on murine immune responses. Benef. Microbes.

[122] Dimidi E., Cox S.R., Rossi M., Whelan K. (2019). Fermented Foods: Definitions and Characteristics, Impact on the Gut Microbiota and Effects on Gastrointestinal Health and Disease. Nutrients.

[123] Kwon G.H., Lee H.A., Park J.Y., Kim J.S., Lim J., Park C.S., Kwon D.Y., Kim Y.S., Kim J.H. (2009). Development of a RAPD-PCR method for identification of Bacillus species isolated from Cheonggukjang. Int. J. Food Microbiol..

[124] Nam Y.D., Yi S.H., Lim S.I. (2012). Bacterial diversity of cheonggukjang, a traditional Korean fermented food, analyzed by barcoded pyrosequencing. Food Control.

[125] Kim Y.S., Kim M.C., Kwon S.W., Kim S.J., Park I.C., Ka J.O., Weon H.Y. (2011). Analyses of Bacterial Communities in Meju, a Korean Traditional Fermented Soybean Bricks, by Cultivation-Based and Pyrosequencing Methods. J. Microbiol..

[126] Jeong D.Y., Ryu M.S., Yang H.J., Park S. (2021). gamma-PGA-Rich Chungkookjang, Short-Term Fermented Soybeans: Prevents Memory Impairment by Modulating Brain Insulin Sensitivity, Neuro-Inflammation, and the Gut-Microbiome-Brain Axis. Foods.

[127] Jeong D.Y., Daily J.W., Lee G.H., Ryu M.S., Yang H.J., Jeong S.Y., Qiu J.Y., Zhang T., Park S. (2020). Short-Term Fermented Soybeans with Bacillus amyloliquefaciens Potentiated Insulin Secretion Capacity and Improved Gut Microbiome Diversity and Intestinal Integrity To Alleviate Asian Type 2 Diabetic Symptoms. J. Agric. Food Chem..

[128] Boulange C.L., Neves A.L., Chilloux J., Nicholson J.K., Dumas M.E. (2016). Impact of the gut microbiota on inflammation, obesity, and metabolic disease. Genome Med..

[129] Lozupone C.A., Stombaugh J.I., Gordon J.I., Jansson J.K., Knight R. (2012). Diversity, stability and resilience of the human gut microbiota. Nature.

[130] Giguere N., Nanni S.B., Trudeau L.E. (2018). On Cell Loss and Selective Vulnerability of Neuronal Populations in Parkinson’s Disease. Front. Neurol..

[131] Surmeier D.J., Obeso J.A., Halliday G.M. (2017). Selective neuronal vulnerability in Parkinson disease. Nat. Rev. Neurosci..

[132] Holmqvist S., Chutna O., Bousset L., Aldrin-Kirk P., Li W., Bjorklund T., Wang Z.Y., Roybon L., Melki R., Li J.Y. (2014). Direct evidence of Parkinson pathology spread from the gastrointestinal tract to the brain in rats. Acta Neuropathol..

[133] Ma L.Y., Liu G.L., Wang D.X., Zhang M.M., Kou W.Y., Feng T. (2019). Alpha-Synuclein in Peripheral Tissues in Parkinson’s Disease. ACS Chem. Neurosci..

[134] De Rui M., Inelmen E.M., Trevisan C., Pigozzo S., Manzato E., Sergi G. (2020). Parkinson’s disease and the non-motor symptoms: Hyposmia, weight loss, osteosarcopenia. Aging Clin. Exp. Res..

[135] Levy O.A., Malagelada C., Greene L.A. (2009). Cell death pathways in Parkinson’s disease: Proximal triggers, distal effectors, and final steps. Apoptosis.

[136] Guo J.D., Zhao X., Li Y., Li G.R., Liu X.L. (2018). Damage to dopaminergic neurons by oxidative stress in Parkinson’s disease (Review). Int. J. Mol. Med..

[137] Trist B.G., Hare D.J., Double K.L. (2019). Oxidative stress in the aging substantia nigra and the etiology of Parkinson’s disease. Aging Cell.

[138] Jenner P., Olanow C.W. (1998). Understanding cell death in Parkinson’s disease. Ann. Neurol..

[139] Xu H., Deng R.X., Li E.T.S., Shen J.G., Wang M.F. (2020). Pinosylvin provides neuroprotection against cerebral ischemia and reperfusion injury through enhancing PINK1/Parkin mediated mitophagy and Nrf2 pathway. J. Funct. Foods.

[140] Zhou J.J., Chen M.F., Wu S.J., Liao X.Y., Wang J., Wu Q.P., Zhuang M.Z., Ding Y. (2020). A review on mushroom-derived bioactive peptides: Preparation and biological activities. Food Res. Int..

[141] Louis P., Flint H.J. (2017). Formation of propionate and butyrate by the human colonic microbiota. Environ. Microbiol..

[142] Singh B.P., Vij S. (2018). In vitro stability of bioactive peptides derived from fermented soy milk against heat treatment, pH and gastrointestinal enzymes. LWT–Food Sci. Technol..

[143] Chakraborty A., Banerjee S., Mukherjee B., Poddar M.K. (2020). Calorie restriction improves aging-induced impairment of cognitive function in relation to deregulation of corticosterone status and brain regional GABA system. Mech. Ageing Dev..

[144] Bourassa M.W., Alim I., Bultman S.J., Ratan R.R. (2016). Butyrate, neuroepigenetics and the gut microbiome: Can a high fiber diet improve brain health?. Neurosci. Lett..

[145] Fischer A., Sananbenesi F., Mungenast A., Tsai L.H. (2010). Targeting the correct HDAC(s) to treat cognitive disorders. Trends Pharmacol. Sci..

[146] Stilling R.M., van de Wouw M., Clarke G., Stanton C., Dinan T.G., Cryan J.F. (2016). The neuropharmacology of butyrate: The bread and butter of the microbiota-gut-brain axis?. Neurochem. Int..

[147] Srivastav S., Neupane S., Bhurtel S., Katila N., Maharjan S., Choi H., Hong J.T., Choi D.Y. (2019). Probiotics mixture increases butyrate, and subsequently rescues the nigral dopaminergic neurons from MPTP and rotenone-induced neurotoxicity. J. Nutr. Biochem..

[148] Kowalski K., Mulak A. (2019). Brain-Gut-Microbiota Axis in Alzheimer’s Disease. J. Neurogastroenterol. Motil..

[149] Dinan T.G., Cryan J.F. (2017). Gut instincts: Microbiota as a key regulator of brain development, ageing and neurodegeneration. J. Physiol..

[150] Quigley E.M.M. (2017). Microbiota-Brain-Gut Axis and Neurodegenerative Diseases. Curr. Neurol. Neurosci. Rep..

[151] Vogt N.M., Kerby R.L., Dill-McFarland K.A., Harding S.J., Merluzzi A.P., Johnson S.C., Carlsson C.M., Asthana S., Zetterberg H., Blennow K. (2017). Gut microbiome alterations in Alzheimer’s disease. Sci. Rep..

[152] Pistollato F., Cano S.S., Elio I., Vergara M.M., Giampieri F., Battino M. (2016). Role of gut microbiota and nutrients in amyloid formation and pathogenesis of Alzheimer disease. Nutr. Rev..

[153] Pistollato F., Iglesias R.C., Ruiz R., Aparicio S., Crespo J., Lopez L.D., Manna P.P., Giampieri F., Battino M. (2018). Nutritional patterns associated with the maintenance of neurocognitive functions and the risk of dementia and Alzheimer’s disease: A focus on human studies. Pharmacol. Res..

[154] Yang H.J., Kwon D.Y., Kim H.J., Kim M.J., Jung D.Y., Kang H.J., Kim D.S., Kang S., Moon N.R., Shin B.K. (2015). Fermenting soybeans with Bacillus licheniformis potentiates their capacity to improve cognitive function and glucose homeostaisis in diabetic rats with experimental Alzheimer’s type dementia. Eur. J. Nutr..

[155] Hsu R.L., Lee K.T., Wang J.H., Lee L.Y.L., Chen R.P.Y. (2009). Amyloid-Degrading Ability of Nattokinase from Bacillus subtilis Natto. J. Agric. Food Chem..

[156] Novik G., Savich V. (2020). Beneficial microbiota. Probiotics and pharmaceutical products in functional nutrition and medicine. Microb. Infect..

[157] Cogliati S., Clementi V., Francisco M., Crespo C., Arganaraz F., Grau R. (2020). Bacillus Subtilis Delays Neurodegeneration and Behavioral Impairment in the Alzheimer’s Disease Model Caenorhabditis Elegans. J. Alzheimers Dis..

